# Spontaneous Coronary Arterial Dissection in a Young Female in the Postpartum Period: A Case Report From Sub-Saharan Africa

**DOI:** 10.7759/cureus.39507

**Published:** 2023-05-25

**Authors:** Maureen Wahinya, Morgan M Ngunjiri, Zahid Khan

**Affiliations:** 1 Internal Medicine, Kenyatta University Teaching, Referral and Research Hospital, Nairobi, KEN; 2 Acute Medicine, Mid and South Essex NHS Foundation Trust, Southend-on-Sea, GBR; 3 Cardiology, Barts Heart Centre, London, GBR; 4 Cardiology and General Medicine, Barking, Havering and Redbridge University Hospitals NHS Trust, London, GBR; 5 Cardiology, Royal Free Hospital, London, GBR

**Keywords:** heart failure with reduced ejection fraction, high troponin-t, st-elevation myocardial infarction (stemi), coronary artery angiogram, post partum, scad and fmd, scad types, multivessel scad, scad in pregnancy, scad management

## Abstract

Spontaneous coronary arterial dissection (SCAD) has become an important cause of acute coronary syndrome (ACS) and sudden cardiac death, particularly in young women, without classic atherosclerotic cardiovascular risk factors. Missed diagnosis is common due to a low index of suspicion in these patients. Here, we present a case of a 29-year-old African female in the postpartum period who presented with a two-week history of heart failure symptoms and acute onset chest pain. An electrocardiogram showed ST-segment elevation myocardial infarction (STEMI) with elevated high-sensitivity troponin T. Echocardiography on admission revealed an ejection fraction of 40% with septal hypokinesia. Coronary angiography showed multivessel dissection with type 1 SCAD in the left circumflex artery and type 2 SCAD in the left anterior descending artery. The patient was managed conservatively, and angiographic healing of SCAD together with normalization of the left ventricular systolic dysfunction was seen after four months. SCAD should always be in the differential diagnosis of any peripartum patient who presents with ACS and lacks the typical atherosclerotic risk factors. Accurate diagnosis and appropriate management are paramount in such cases.

## Introduction

Spontaneous coronary arterial dissection (SCAD) is a potentially fatal disease that occurs without atherosclerosis, trauma or iatrogenic causes [1]. Once thought to be a rare condition affecting younger patients, particularly women, recent data shows that SCAD is more frequent than previously thought [2]. Although the true prevalence of SCAD is unknown, particularly in sub-Saharan Africa (SSA), it is now increasingly recognized as an important cause of acute coronary syndrome (ACS) accounting for 1%-4% of all ACS cases, and 35% of all ACS cases in women under 50 years of age [2]. It remains the commonest cause of pregnancy-associated myocardial infarction (MI), accounting for approximately 43% of cases [3]. Some of the potential predisposing factors include fibromuscular dysplasia, pregnancy, hormone therapy, high parity (>four births), systemic inflammatory diseases and connective tissue diseases [2]. Missed diagnoses are common due to a low suspicion index in young patients with no atherosclerotic cardiovascular risk factors. Here, we present a case of pregnancy-associated SCAD (P-SCAD) in a 29-year-old African female with no atherosclerotic cardiovascular risk factors.

## Case presentation

A 29-year-old woman, para 3+0, three weeks postpartum, was referred to our hospital with a working diagnosis of acute ST-segment elevation myocardial infarction (STEMI) and bronchopneumonia. She presented with a two-week progressive history of lower limb oedema, dyspnoea, nocturnal cough and fever. She was graded as New York Heart Association (NYHA) class IV for her dyspnoea. The symptoms had been preceded by progressively worsening, non-radiating left-sided dull chest pain. She had no identifiable atherosclerotic risk factors, and was a lifelong non-smoker and a teetotaller. She had a normal body mass index (BMI), was young, had no known comorbid conditions and had no family history of heart disease or sudden cardiac death.

**Figure 1:**
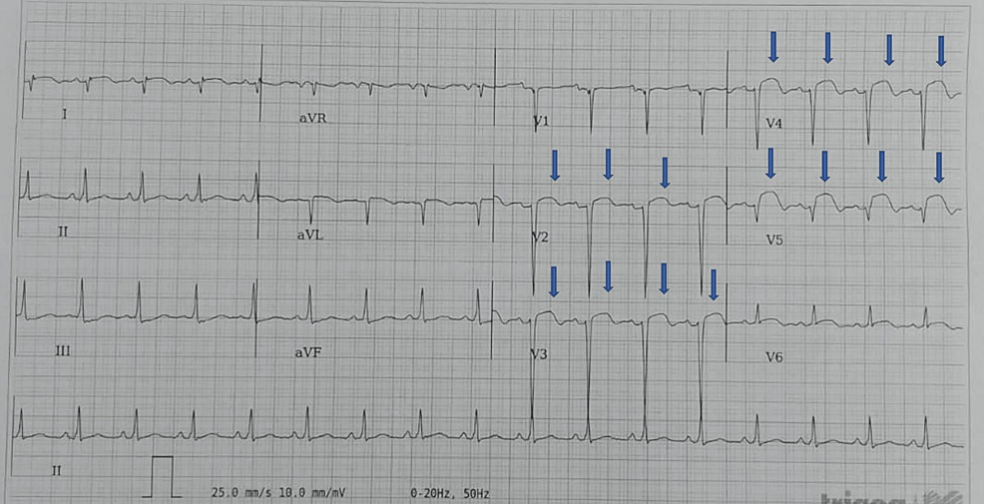
Electrocariogram showing ST segment elevation in leads V2-V5 (arrows)

Initially, she assumed childbirth to be the cause of her symptoms and did not seek medical assistance for up to two weeks. However, when symptoms did not improve, she presented to the emergency department to seek medical help. On examination, she was in severe respiratory distress requiring oxygen supplementation at 12 litres per minute via a non-rebreather mask to maintain oxygen saturation above 94%. Her blood pressure was 145/87 mmHg and pulse was 102 beats per minute. A systemic examination revealed bilateral basal coarse lung crepitations and a granulating superior upper midline abdominal caesarean-section scar, without signs of infection. An electrocardiogram showed sinus tachycardia and right axis deviation with ST segment elevation (STEMI) in leads V3, V4 and V5 (Figure 1).

High-sensitivity troponin T was found to be elevated at 26698 ng/l (0-40 ng/l), creatine kinase myoglobin binding (CKMB) was elevated at 22.85 ng/ml (0-4.9 ng/ml), and N-terminal pro B atrial natriuretic peptide level was 4954 pg/ml on the day of admission and 465 pg/ml on day 4 of hospitalization. D-dimer was negative for pulmonary embolism. Her lipid profile and clotting tests were normal, and hepatitis and viral screening was negative. A transthoracic echocardiogram (TTE) showed non-dilated cardiomyopathy with septal hypokinesia and a left ventricular ejection fraction (LVEF) of 42% (Video 1).

**Video 1:** Transthoracic echocardiogram showing non-dilated cardiomyopathy with septal hypokinesia and an impaired left ventricular ejection fraction

A high-resolution computed tomography (CT) scan of the chest showed features of pulmonary oedema with atypical pneumonia. She was managed for acute pulmonary oedema secondary to evolved acute coronary syndrome and bronchopneumonia. She was started on aspirin, clopidogrel, atorvastatin, intravenous furosemide and intravenous antibiotics. The patient underwent invasive coronary angiography on day 3 of admission that showed type 2 SCAD of the left anterior descending artery (LAD) with terminal flow of TIMI 1 and type 1 SCAD of the left circumflex artery with good distal flow (Video 2).

**Video 2:** Coronary angiogram showing type 2 SCAD of the left anterior descending artery and type 1 SCAD of the left circumflex artery SCAD, spontaneous coronary arterial dissection

A decision to manage her conservatively with medical therapy was made. During her hospital stay, she recovered and was discharged on isosorbide mononitrate 20 mg twice a day (BD), carvedilol 12.5 mg BD, sacubitril/valsartan 50 mg BD, Aldactone 25 mg once a day (OD), furosemide 20 mg OD, empagliflozin 10 mg OD, aspirin 75 mg OD and clopidogrel 75 mg OD. Repeat TTE after four months revealed improvement in the LVEF at 60%, and coronary angiography revealed complete resolution of the SCAD (Videos 3, 4). She is currently only on bisoprolol and aspirin; rest of her medications were stopped at three to four months following the recovery of LV function.

**Video 3:** Repeat echocardiogram showing recovery of left ventricular function

**Video 4:** Repeat coronary angiogram showing a complete resolution of the spontaneous coronary artery dissection

## Discussion

SCAD is the most common cause of MI among pregnant women or women in their postpartum period [3]. Most cases of P-SCAD occur in the third trimester or within the first four weeks postpartum, with some cases being reported in early pregnancy and in lactating women outside the postpartum period [4]. In the United States, it is estimated that for every 16,000 pregnancies, one pregnancy would be complicated by MI and a quarter of these MI cases are a result of SCAD [5,6]. In a more recent nationwide US survey among pregnant women and women in the puerperium period, the prevalence of SCAD was reported as 1.81 cases per 100,000 pregnancies [7].

Hormonal changes occurring during pregnancy have been implicated in the architectural alteration of the coronary arterial walls leading to a weakening of the vessel walls, hence making them vulnerable to rupture [3]. The heightened risk of SCAD seen in multiparous women further emphasizes this theory of cumulative arterial wall changes over several pregnancies [8,9]. The underlying mechanism of myocardial injury from SCAD is coronary artery obstruction caused by an intramural hematoma. The pathophysiology of SCAD is not fully elucidated; however, two theories have been proposed. The first one suggests that an intimal tear leads to coronary artery dissection resulting in a false lumen filled with intramural hematoma with consequent obstruction of the true lumen and subsequent myocardial infarction and ischaemia. The second theory proposes that the initiating event is spontaneous bleeding from the vasa vasorum that propagates into the medial wall forming an intramural hematoma that sometimes ruptures into the true lumen [10].

Patients with SCAD present with symptoms similar to those of atherosclerotic ACS in addition to elevated troponin levels. The most common presentation is angina-like chest pain, with some patients presenting with heart failure, ventricular arrhythmias or sudden cardiac death [9]. Data from research studies show that ST-segment elevation MI is the initial presentation in approximately 26%-87% of SCAD patients, while non-ST-segment elevation MI is the initial presentation in about 13%-69% of the patients [11-15]. Patients with P-SCAD tend to be younger, with an average age of 33-38 years [7]. In addition, they are more likely to have STEMI, LV systolic dysfunction at presentation, and dissection affecting the left main coronary artery or multivessel dissections [8].

Our patient fit within the profile of P-SCAD demographics and clinical characteristics as she was multiparous, within the given age range and presented with STEMI in the anterolateral leads, had heart failure with an ejection fraction of 42% and had multivessel dissection affecting the LAD and the left circumflex artery. A high index of suspicion is required to diagnose SCAD because the patient demographics do not conform to the expected phenotype for a high-risk atherosclerosis cardiovascular disease patient.

Several imaging modalities can be used to confirm the diagnosis of SCAD, such as coronary angiography, coronary CT angiography and intracoronary imaging such as optical coherence tomography or intravascular ultrasonography. A major limitation to coronary angiography is that it is two-dimensional and hence it might not directly image the lumen of the arteries. However, despite its inherent limitations, coronary angiography remains the initial imaging modality of choice because it is widely available and relatively affordable in most countries [12].

SCAD can be classified into three types based on the Saw angiographic classification of SCAD [1]. Type 1 is considered the stereotypical appearance with contrast arterial wall staining. This appears as multiple radiolucent lumens on the coronary artery. Although considered the classical appearance for SCAD, it is present in less than 30% of cases. Type 2 is the most common angiographic finding appearing as a long diffuse stenosis bordered by normal-appearing segments proximal and distal to the intramural hematoma. In some cases, the lesion extends distally. Type 3 is a focal or tubular stenosis that mimics atherosclerosis. The lesion is usually less than 20 mm in length.

Although CAD is a leading cause of death in developed countries, it is considered to be rare in sub-Saharan Africa though its incidence and prevalence are on the rise lately. Kengne et al. reported that cardiovascular diseases (CVDs) are more common than previously thought in diabetic patients in SSA; however, the diagnosis and understanding of coronary heart disease has been limited by the inadequacy of resources and shortage of physicians [14]. Nkoke and Luchuo reported two cases where patients with chest pain and epigastric pain were treated for gastritis by the emergency physicians, which was likely due to the lack of resources. They argued that more cases like these were perhaps getting missed due to the lack of resources [13]. Mensah reported that CVD was the eighth leading cause for mortality in SSA and this risk was increasing due to lifestyle changes associated with urbanisation and the epidemiological transition [15]. The INTERHEART Africa study found hypertension, smoking, diabetes, obesity and dyslipidaemia to have an attributable risk of about 90% for acute coronary events [16].

The management of patients with SCAD is mainly conservative, except for patients who present with hemodynamic instability, have involvement of the left main artery, or have ongoing ischaemia despite conservative management [17]. A conservative approach is initially preferred because it has been demonstrated that angiographic healing is evident after four weeks in approximately 90% of SCAD patients [18]. Antiplatelet therapy is recommended in all patients with SCAD. However, due to the paucity of evidence, dual antiplatelet therapy (DAPT) is not generally recommended unless the patient underwent percutaneous coronary intervention in which case the duration of DAPT should be a minimum of one year. Nevertheless, some experts opt for short-term DAPT for one to three months, followed by lifelong aspirin therapy [2]. Anticoagulation is not recommended and should be stopped once a diagnosis of SCAD is made [2]. Beta blockers are the mainstay of treatment in patients with SCAD because they are associated with a lower risk of recurrence and have a clear mortality benefit [19,20]. Angiotensin-converting enzyme inhibitors and angiotensin receptor blockers (ACEI/ARB) are reserved for patients with LV systolic dysfunction. Statins are only indicated in patients with concomitant dyslipidaemia or with other guideline-directed indications. Antianginal medications like calcium channel blockers, nitrates or ranolazine are reserved only for the relief of angina symptoms [2]. Glycoprotein IIb/IIIa inhibitors and thrombolytics are not recommended because of the potential of worsening the intramural hematoma and coronary artery rupture [20].

## Conclusions

Accurate diagnosis, appropriate management and future risk reduction strategies are paramount in managing patients with spontaneous coronary arterial dissection. SCAD should be placed high in the differential diagnosis of young patients without atherosclerosis cardiovascular risk factors, presenting with symptoms similar to acute coronary syndrome. Similarly, pregnancy-associated SCAD should always be strongly considered in the differential diagnosis of any peripartum patient presenting with MI. Raising awareness among clinicians regarding the same, particularly in sub-Saharan Africa, is essential.
